# Structural and Functional Insights into GluK3-kainate Receptor Desensitization and Recovery

**DOI:** 10.1038/s41598-019-46770-z

**Published:** 2019-07-16

**Authors:** Jyoti Kumari, Rajesh Vinnakota, Janesh Kumar

**Affiliations:** Laboratory of Membrane Protein Biology, National Centre for Cell Science, NCCS Complex, S. P. Pune University, Maharashtra Pune, 411007 India

**Keywords:** Ion channels in the nervous system, X-ray crystallography, Cryoelectron microscopy

## Abstract

GluK3-kainate receptors are atypical members of the iGluR family that reside at both the pre- and postsynapse and play a vital role in the regulation of synaptic transmission. For a better understanding of structural changes that underlie receptor functions, GluK3 receptors were trapped in desensitized and resting/closed states and structures analyzed using single particle cryo-electron microscopy. While the desensitized GluK3 has domain organization as seen earlier for another kainate receptor-GluK2, antagonist bound GluK3 trapped a resting state with only two LBD domains in dimeric arrangement necessary for receptor activation. Using structures as a guide, we show that the N-linked glycans at the interface of GluK3 ATD and LBD likely mediate inter-domain interactions and attune receptor-gating properties. The mutational analysis also identified putative N-glycan interacting residues. Our results provide a molecular framework for understanding gating properties unique to GluK3 and exploring the role of N-linked glycosylation in their modulation.

## Introduction

Ionotropic glutamate receptors (iGluRs) mediate the majority of fast excitatory neurotransmission at the chemical synapses in the central nervous system (CNS). Due to this pivotal role, their dysfunction is implicated in a wide range of neurological disorders such as schizophrenia, neuro-excitatory disorders, epilepsy, neurodegenerative, developmental disorders, neuropathic pain etc^1^. The iGluR family is broadly classified into four classes such as α-amino-3-hydroxy-5-methyl-4-isoxazolepropionic acid receptor (AMPA), kainate (KA), N-methyl-D-aspartate (NMDA) and orphan delta receptors. AMPA, KA, and NMDA receptors form tetrameric cation conductive channels that are activated by presynaptically released glutamate resulting in an influx of sodium and calcium ions leading to membrane depolarization^2^.

Although, all the iGluRs share similar domain organization, membrane topology and high sequence similarity, they have distinct pharmacological, physiological and biophysical properties^3^. In contrast to the AMPA and NMDA receptors that are primarily localized on the post-synaptic membrane and are directly involved in neurotransmission, increasing evidence has shown that KA receptors are expressed at both the presynaptic and postsynaptic sites. They play a crucial role in the regulation of synaptic networks by modulating neurotransmitter release at the presynaptic sites and by participating in membrane depolarization at postsynapses^4^. KA receptors are abundantly expressed in hippocampus and cerebellum; display characteristic slow kinetics, are mainly involved in long-term memory formation and motor control^5,6^. They are divided into two families consisting of the “low-affinity” glutamate binding subunits (GluK1-GluK3) that form functional homomeric ion channels and “high-affinity” subunits (GluK4 and GluK5) that form functional receptors only on assembly with “low-affinity” subunits^7–9^.

Despite essential role of GluK3 receptors at the presynaptic sites in the hippocampal mossy fibre synapse^10^, it is one of the least studied KA subunits with respect to structure-function analysis. Owing to this, structural insights into unique functional properties of GluK3 receptors like faster desensitization rates of partially bound homomeric receptors^11^, activation by higher glutamate concentrations and potentiation by zinc^12^ is lacking. However, crystal structures of GluK3 ligand binding (LBD)^12,13^ and amino-terminal domains (ATD)^14,15^ has been reported but full-length structures of GluK3 are still elusive.

Further, iGluRs including GluK3, are extensively N-glycosylated which likely imparts an additional level of regulation of their functions. These glycans have been reported to affect the trafficking, gating properties^16–18^ and assembly of iGluRs^19–24^. They likely affect receptor functions by interactions with other synaptic proteins or by mediating inter- and intra-domain interactions in iGluRs^19^ affecting receptor functionality^20^. However, detailed insights into these mechanisms is lacking.

In order to understand the conformational changes underlying GluK3 functions in the context of full-length tetrameric receptors and to explore the role of N-glycans in modulating their functions, we expressed and purified rat GluK3 receptors from baculovirus-infected HEK293 GnTI^−^ cells; trapped them in agonist and antagonist bound states and determined their structures via single particle cryo-electron microscopy. We also solved crystal structure of GluK3 ligand binding domain in complex with agonist SYM. In addition, utilizing these full-length GluK3 structures and electrophysiological assays, we explored the involvement of N-glycosylation sites in the receptor function. Our results shed light upon the conformational changes underlying GluK3 receptor transition between desensitized and resting states and the importance of N-glycosylation in modulating its functions.

## Results

### Receptor purification and structure determination

**Figure 1:**
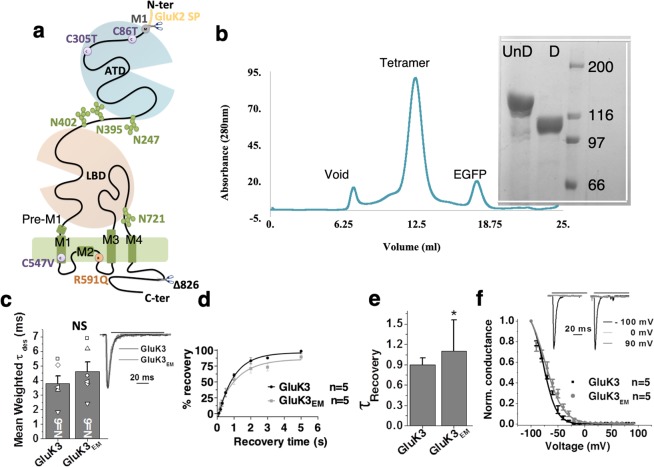
Construct design and optimization of GluK3 receptor for structural studies. (**a**) Schematic representation of GluK3_EM_ construct. Amino-terminal domain (ATD), ligand binding domain (LBD), and transmembrane domain (TMD) are shown along with the mutations made for construct optimization. GluK2 signal peptide instead of native GluK3 signal peptide and C-terminal truncation at residue 826 led to improved protein expression. Point mutations of free cysteines C86T, C305T in ATD and C547V in TM domain are shown in violet while R591Q mutant in TM is highlighted in orange. N-glycans at the ATD-LBD interface and the LBD-TM interface are also shown. (**b**) The SEC profile demonstrates monodisperse tetrameric receptor peak eluted from the Superose 6 column and SDS-PAGE analysis of undigested (UnD) and thrombin digested (D) GluK3_EM_ is shown. SDS-PAGE gel has been cropped to show bands above 66 kDa and is indicated by white lines. Panels c–f show electrophysiological studies of the wild type and GluK3_EM_ construct with very similar rates for desensitization (**c**); recovery from desensitization (**d-e**) and indistinguishable normalized conductance measured from −100 to +100 mVolts (**f**). Representative rectification index at −100 mV, 0 mV and +90 mV are shown for wild-type (black) and GluK3_EM_ (grey). Amplitudes of normalized currents for wild-type and GluK3_EM_ evoked on the application of 30 mM glutamate is shown in inset **c**.

Multiple mutations and changes were necessary in the wild type GluK3 receptor to improve its expression and stability in heterologous expression system as elucidated in Fig. 1a and Supplementary Fig. 1. The final construct referred to as GluK3_EM_ (Fig. 1a) henceforth was optimized using the fluorescence-detection size exclusion chromatography (FSEC)^25^ and had a symmetrical profile corresponding to tetrameric receptors on size exclusion chromatography (Fig. 1b). GluK3_EM_ exhibited gating properties similar to wild type (WT) receptor as validated by whole-cell patch clamp recordings in HEK293T cells. It had comparable rectification properties, rates of entry into desensitization and recovery from the desensitized state (Fig. 1c–f, Table 1) consistent with previous reports^11^.

**Table 1:** Electrophysiological assays for the wild type and mutant GluK3 receptors.

N-Glycan knockout	Mutation Name	Desensitization (Mean weighted, ms)	Dose-response (EC 50) mM (95% CI)	Recovery time constant (S) (95% CI)	Rise Time (ms)
	**GluK3**	3.8 ± 0.5 (n = 6)	9.5 (n = 7) (8.4 to 10.7)	0.9 (n = 5) (0.8 to 1.2)	1.3 ± 0.1 (n = 7)
	**GluK3**_**EM**_	4.6 ± 0.7 (n = 6) P = 0.3743		1.1 (n = 5) (0.6 to 2.4)	
**GluK3 N-Glycan Mutations**					
N 247	**T249A**	3.7 ± 0.4 (n = 4) P = 0.8900	7.2 (n = 5) (6.5 to 7.9) *P = 0.0142	0.5 (n = 5) (0.3 to 1.0) P = 0.0870	1.3 ± 0.1 (n = 5)
N 395	**T397A**	3.2 ± 0.2 (n = 10) P = 0.2130	3.7 (n = 5) (3.7 to 7.2) ****P < 0.0001	0.8 (n = 5) (0.4 to 1.9) P = 0.8067	1.1 ± 0.1 n = 6)
N 402	**S404A**	4.5 ± 0.3 (n = 6) P = 0.2576	10.3 (n = 5) (9.3 to 11.4) P = 0.7602	0.5 (n = 4) (0.4 to 0.7) *P = 0.0201	1.4 ± 0.1 (n = 6)
N247/N395	**T249A/T397A**	4.3 ± 0.2 (n = 11) P = 0.2861	6.5 (n = 4) (6.0 to 7.0) **P = 0.0022	0.6(n = 6) (0.4 to 0.8) P = 0.0694	1.7 ± 0.1 (n = 6)
N247/N402	**T249A/S404A**	4.1 ± 0.2 (n = 5) P = 0.6184	5.3 (n = 5) (4.9 to 5.6) ****P < 0.0001	0.3 (n = 5) (0.2 to 0.4) ***P = 0.0007	
N395/N402	**T397A/S404A**	4.0 ± 0.4 (n = 9) P = 0.7587	5.7 (n = 5) (5.3 to 6.1) ****P < 0.0001	0.5 (n = 6) (0.4 to 0.6) **P = 0.0047	1.2 ± 0.1 (n = 6)
**Potential N-Glycan interacting residues**					
	**E239L**	4.0 ± 0.2 (n = 5) P = 0.7388		0.3 (n = 4) (0.2 to 0.5) **P = 0.0028	1.6 ± 0.1 (n = 5)
	**R242E**	3.9 ± 0.2 (n = 6) P = 0.8564		0.8 (n = 5) (0.5 to 1.6) P = 7463	
	**Y243F**	4.4 ± 0.7 (n = 5) P = 0.4929		0.6 (n = 4) (0.5 to 0.8) *P = 0.0465	1.4 ± 0.1 (n = 5)
	**R242E/Y243F**	6.0 ± 1.3 (n = 5) P = 0.1241		4.0 (n = 5) (2.5 to 7.2) *P = 0.0328	1.5 ± 0.3 (n = 4)
**Desensitization ring residues**					
	**D672R**	3.5 ± 0.1 (n = 5) P = 0.6052		0.7 (n = 5) (0.6 to 0.9) P = 0.1554	1.1 ± 0.2 (n = 6)

All values are rounded off to the single decimal place. Errors are reported as SEM. Statistical significance is indicated as *P < 0.05, **P < 0.01, ***P < 0.001, ****P < 0.0001 for comparisons between wild type GluK3 and various mutants.

**Figure 2:**
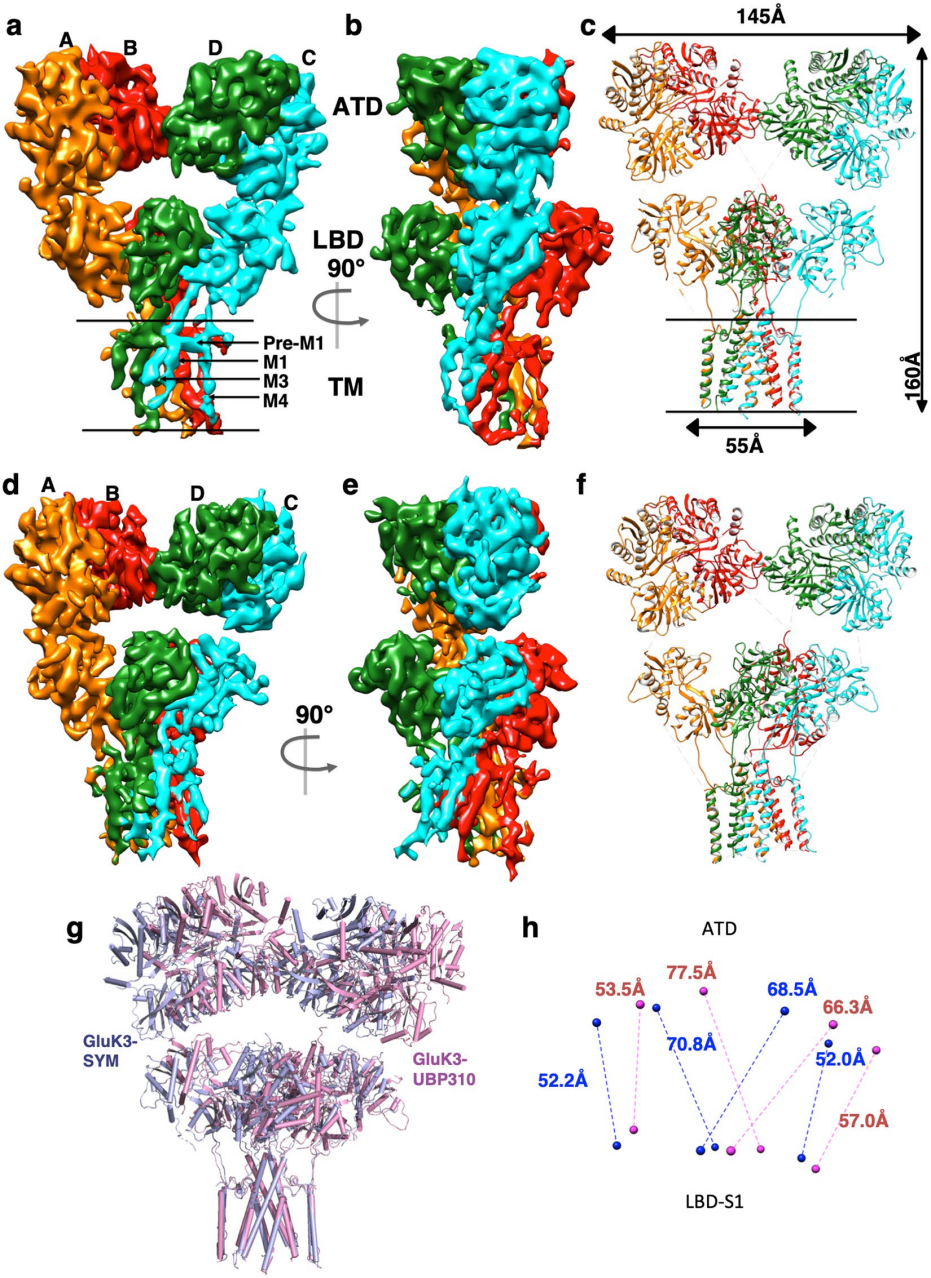
Cryo-EM density map and fitted model for the agonist and antagonist bound GluK3. Segmented and colored cryo-EM density map at 7.4 Å for agonist 2S,4R-4-methyl glutamate-bound (**a**–**c)** and at 7.7 Å for antagonist UBP310 bound GluK3_EM_ **(d–f**), (**a**) and (**d**) show the front view of receptor, perpendicular to the overall two-fold axis of molecular symmetry with each subunit colored uniquely; (**b**,**e**) are 90° rotated views of (**a**,**b**); (**c**,**f**) show the fitted atomic model colored to represent four receptor subunits as in (**a,b**) and (**d**,**e**). Panels g,h show reorientation of the extracellular domains during the transition from agonist-bound to antagonist bound state. (**g**) Shows front view (perpendicular to the global 2-fold axis of symmetry) of the full-length GluK3 receptor structure in complex with SYM (in cyan), superposed on the UBP310 structure (magenta) by aligning the TMD regions. Distances between the centre of masses, shown as spheres between ATD and LBD-S1 lobe of each subunit in SYM (blue) and UBP310 (red) bound receptor is measured and indicated in panel h.

To understand the structural basis of the receptor rearrangements during its gating cycle, we elucidated the GluK3_EM_ structure in the desensitized and resting/closed state using single particle cryo-EM. These states were captured in the presence of agonist 2S, 4R-4-methyl glutamate (SYM)^26^ and antagonist UBP310^27^ respectively (Supplementary Fig. 2). Reference-free 2D classification obtained after cryo-EM data processing for both the complexes showed good distribution of various receptor orientations as well as identifiable external features resembling glutamate receptor (Supplementary Fig. 3a,b). Various steps of EM data processing and 3D reconstruction of the complexes reveals similar tetrameric three-layered receptor architecture as previously observed for GluA2^28,29^ and GluK2^30,31^ (Fig. 2; Supplementary Fig. 4a,b). The final density map of the closed and desensitized states has an estimated resolution of ~7.7 Å and 7.4 Å as per 0.143 FSC gold standard respectively (Supplementary Figs 5 and 6). Details of cryo-EM data collection and refinement are shown in Table 2. Our EM maps show distinct features for the ATD and LBD domains except for the linkers connecting S1-M1 and M3-S2 domains that are poorly resolved (Supplementary Fig. 7). Co-ordinates of previously reported GluK3 ATD (PDB ID: 3OLZ)^14^ and LBD-SYM complex structure determined in this study were fitted into EM density map for generating a model of the desensitized receptor. Model for TM domain was generated by threading GluK3 sequence onto the GluK2 TM domain (PDB ID: 5KUF)^31^. For GluK3-UBP310, an extended LBD model was generated by threading GluK3 sequence onto the GluK2 LBD-LY complex (PDB ID: 5CMK)^31^. These individual models were rigid body fitted into EM maps of GluK3-SYM and GluK3-UBP310 to generate tetrameric receptor models in UCSF-Chimera^32^ and subsequently subjected to multiple rounds of real space refinement as implemented in Phenix software suite^33^.

**Table 2:** Cryo-EM data collection, refinement and validation statistics.

	GluK3-2S,4R-4-methyl glutamate	GluK3-UBP310
**Data collection and processing**		
PDB ID	6JFY	6JFZ
EMDB ID	9821	9822
Microscope	Titan Krios	Titan Krios
Voltage (kV)	300	300
No. of Micrographs	719	1693
Camera	Falcon 3	Falcon 3
Mode of recording	Super-resolution counting	Super-resolution counting
Exposure time (seconds)	60	60
Electron exposure (e–/Å^2^)	16.73	17.27
Defocus range (μm)	1.5–3.3	1.5–3.3
Pixel size (Å)	1.38	1.41
Symmetry	C1	C1
Initial particle images (no.)	77,276	437,986
Final particle images (no.)	9,730	27,194
Map resolution (Å) FSC threshold	7.4 0.143	7.6 0.143
**Refinement**		
Initial model used (PDB code)	3OLZ (ATD), 6JMV(LBD), 5KUF(TM)	3OLZ (ATD), 5CMK(LBD), 5KUF(TM)
Model resolution (Å) FSC threshold Map-to model fit, CC_mask	7.5 0.5 0.85	7.7 0.5 0.86
**Model composition**		
Non-hydrogen atoms Protein residues	22875 2877	22948 2869
**R.m.s. deviations**		
Bond lengths (Å) Bond angles (°)	0.007 1.2	0.006 1.119
**Validation**		
MolProbity score Clashscore	2.05 11.77	2.14 15.2
Ramachandran plot		
Favored (%) Allowed (%) Disallowed (%)	92.5 7.4 0.1	93.0 7.0 0.0

### Desensitized and closed state GluK3 receptor structure

The desensitized and closed-state structures have a similar three-layered arrangement of the ATD, LBD and TM domains as reported earlier for other iGluRs. Receptor assembly is mainly mediated by ATD as a dimer of the dimer (AB and CD) and then undergoes domain swapping at the LBD layer (BC and AD) as observed for AMPA^28,29,34^ and kainate^30,31^ receptors.

**Figure 3:**
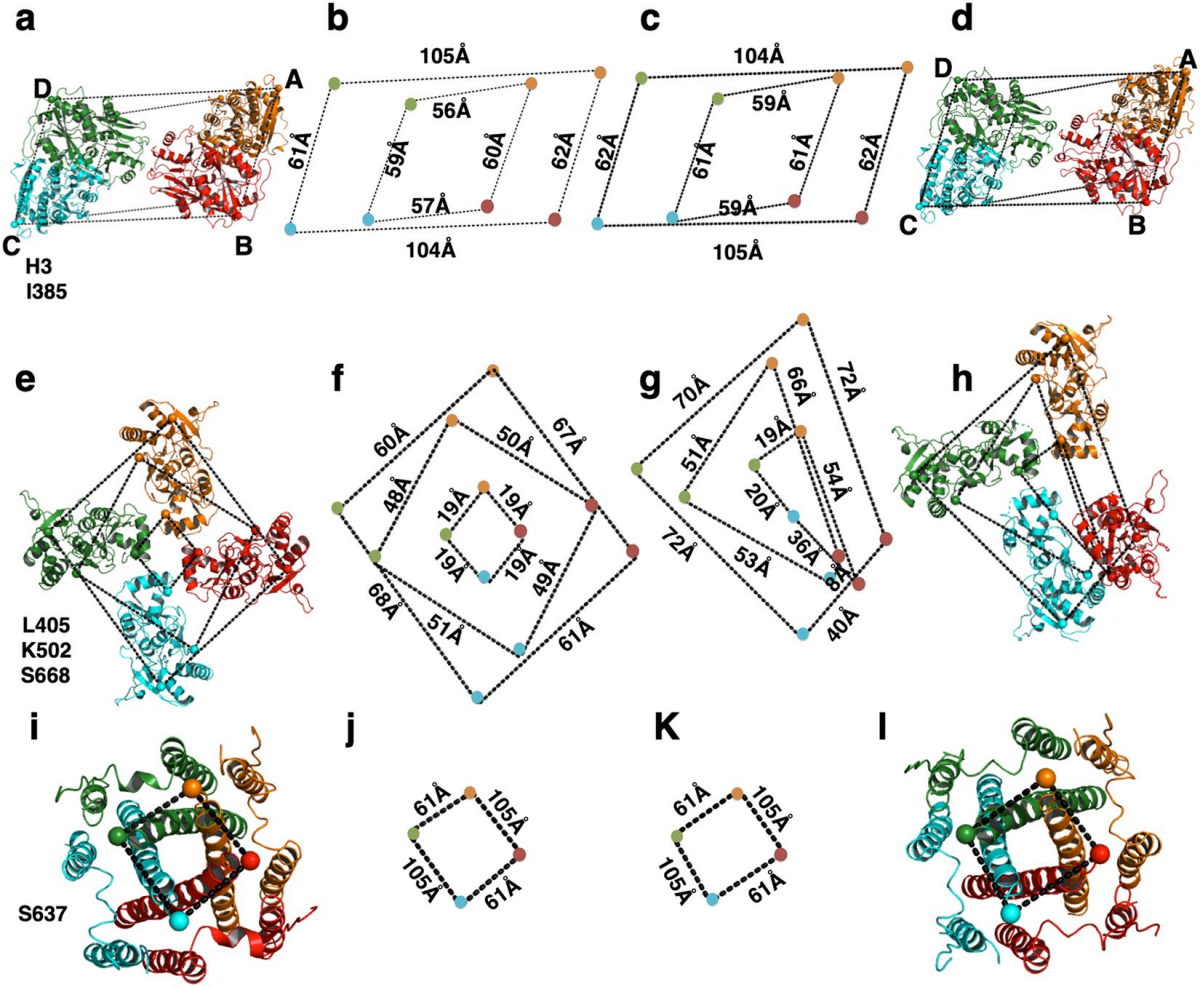
Structural comparison of extracellular and TM domains in SYM and UBP310 bound GluK3_EM_. (**a–d**) Top views of ATD (upper panel); (**e–h**) LBD (middle panel) and (**i–l**) TMD layers (lower panel) are shown. Cα - Cα distances between subunits for selected positions are measured and indicated. Colored dots connected by dashed lines identify the locations of His 3 and Ile 382 at the top and base of the ATD and indicate very similar parallelograms formed for both SYM (**a,b**) and UBP310 (**c,d**) bound forms. Panels e–h show positions of Cα atoms for Leu 405, Lys 502 and Ser 668 indicating the top, middle, and base of the LBD connected by dashed lines. Symmetric parallelograms are formed at all levels of LBD for SYM (**e,f**), while asymmetric trapezoids are observed in UBP310 bound state shown in (**g**,**h**). Top view of the TM domains with Ser 637 connected by dashed lines is shown in (**i**,**j**) (SYM) and (**k**,**l**) (UBP310).

The ATD domains in both GluK3-SYM and GluK3-UBP310 complex have a N-shaped arrangement with an apparent 2-fold symmetry at dimer and dimer of dimers interface, while the TM domains exhibit 4-fold symmetry (Figs 2 and 3a–d; Supplementary Fig. 8). The recently reported O-shaped arrangement of the ATD layer in AMPA receptor was not observed^35^ in our analysis. Consistent with this, the parallelogram formed by joining Cα atoms of His 3 from each subunit at top and Ile 385 at the base has a similar dimension in the desensitized and closed state (Fig. 3b,c). This also suggests that there are no major structural changes at the ATD layer in the desensitized and resting/closed states. Further, the resolution of our EM maps is adequate to (Supplementary Fig. 7) show unambiguously that in both desensitized and closed state the ion channel adopts a closed pore configuration, where the M3 helices are arranged in a crossed bundle assembly with the pre-M1 helices wrapped around the channel. Thus, the TM domains in both cases form a similar parallelogram with dimensions of ~61 Å × 105 Å on joining Cα atoms of Ser 637 from all the subunits (Fig. 3i–l).

In contrast to ATD and TM domains, comparison of the SYM and UBP310 bound structures show major conformational changes at the LBD layer. LBD in GluK3-SYM bound state adopts an apparent 4-fold symmetry (Fig. 3e) and is similar to that reported for GluK2 receptors^30,31^ (Supplementary Fig. 9). Thus, the progressively reducing parallelograms formed by joining Cα atoms of Leu 405, Lys 502 and Ser 668 at the top, middle and at the base of LBD have dimensions indicative of a symmetric arrangement of both upper and lower LBD lobes (Fig. 3e,f). In the GluK3-UBP310 complex, however, we observe previously unseen asymmetric arrangement for kainate receptors trapped in a closed state. Interestingly, subunits B and C form the LBD dimer characteristic of an antagonist-bound form seen in both GluA2 and GluK2 receptors; while the subunits A and D are separated and exist in a desensitized-like state but with an extended LBD cleft (Fig. 3h). Due to such arrangement, joining of Cα atoms of Leu 405, Lys 502 and Ser 668 at the top, middle and at the base of LBD forms an asymmetric trapezium of reducing dimensions shown in Fig. 3g,h. It is interesting to note that all the four LBD domains are in an extended cleft conformation characteristic of an antagonist-bound state in UBP310 complex; whereas SYM bound density map reveals LBD ‘clamshell’ closure (Supplementary Fig. 10). Our cryo-EM analysis does not reveal any 2D or 3D class similar to resting state observed in GluK2 and GluA2, where LBDs are arranged as two dimers with 2-fold symmetry, the trapped structure likely represents a stable conformation. However, it is possible that we have trapped an intermediate between the desensitized and resting state. Besides, the LBD arrangement in GluK3-UPB310 complex is similar to that observed in GluA2 complex with partial agonist fluorowillardine^29^ but with LBD clamshell in an extended configuration. Hence it is likely that the resting state in GluK3 with the LBDs coupled as two dimers may not be as stable as in AMPA and kainate receptors of known structures. Interestingly, GluK3 residue D759 from one protomer faces D730 from other protomer on LBD dimer formation. This close apposition of two similarly charged residues likely destabilizes the dimer interface by electrostatic repulsion. Binding of Zn ion at this site is shown to increase the dimer stability and potentiate GluK3 currents^12^. The resting state conformation observed in our structure might contribute to low glutamate sensitivity of GluK3 owing to faster entry into the desensitized state and reduced transduction of ligand-binding to the pore domain.

In resting state, the ligand binding is uncoupled from the transmembrane domain as strain on the linkers connecting LBD to TM domains are relaxed by the extended conformation of antagonist bound LBD (even while they are in dimeric configuration). In the desensitized state, the LBD-TM linker strain due to bound agonist is relaxed via rearrangements of LBD from a dimer of dimer scheme to a pseudo-4 fold symmetric arrangement, where the LBD dimers are disrupted (Fig. 2). In concordance with this, the TM domains in both the structures adopt similar closed pore configuration.

### Transitions from desensitized to resting/closed state require substantial conformational changes in LBD and the LBD-TM linkers

**Figure 4:**
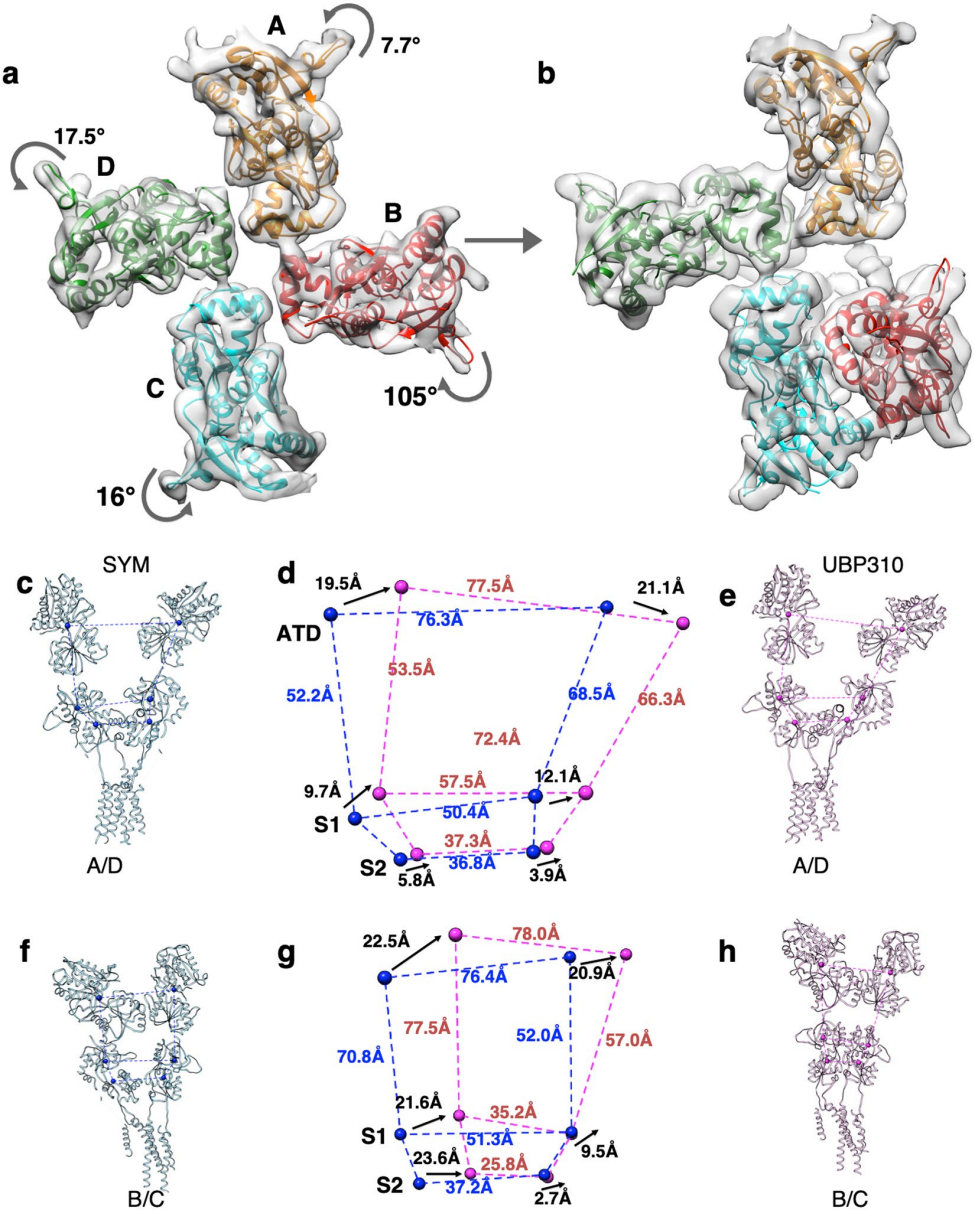
Conformational changes during the transition from desensitized to resting state. (**a,b**) Top views of the segmented density maps for LBD fitted with coordinates shown in ribbon in (**a**) agonist-bound desensitized and (**b**) antagonist-bound resting state. Structural changes in an LBD tetramer assembly underlying the transition from desensitized to resting state are depicted. Degree and direction of LBD rotations are measured and indicated. Panels c–h show subunits A/D and B/C for SYM and UBP310 bound receptor aligned on TM domains. Distances from the centre of mass (spheres) between subunits for the ATD (top) and LBD S1 & S2 lobes are measured and indicated by dashed lines. Structures were superimposed using main-chain atoms of the TM domains. Panels d & g show superimpositions of the parallelograms formed for the A/D (**d**) and B/C (**h**) subunits. The movements of the COM for domains between SYM and UBP310 structures are measured and indicated in black fonts, while the distances within structures are indicated in blue (SYM) and red (UBP310).

In the gating cycle, post-desensitization, receptor needs to recover to a resting state, where the LBDs couple as 2-dimers for the next cycle of activation. Hence, in a “fully-recovered” receptor, the four LBDs should rearrange into two dimers as observed previously in GluK2^31^ and GluA2^28,29^ closed-state structures to enable coupling of agonist binding with channel opening^36^. To understand this transition for GluK3 receptors, we compared SYM and UBP310 bound structures. It was observed that there is very little change in the conformations of the ATD and TM domains. On the contrary, major changes take place in the LBD layer and in the linkers connecting S1-M1, M3-S2, and S2-M4 as reported earlier for GluA2 and GluK2. Interestingly, ligand binding domains of only subunits B and C return to a dimeric state, while subunits A and D remain in a desensitized–like state (Fig. 4). The LBD-distal subunit B swings clockwise by ~105° in the horizontal plane, while the LBD-proximal subunit C rotates anticlockwise by ~16° to achieve dimeric configuration (Fig. 4a,b). In contrast to this, the LBD of subunit A and D undergo a smaller degree of anti-clockwise rotation by ~7.7° and ~17.5° respectively and hence are unable to achieve a dimeric configuration (Fig. 4a,b). To further analyze structural changes in detail, we aligned both receptors at TM domains and calculated centre of masses (COM) for ATD and S1, S2 lobes of LBDs to measure their displacement in going from SYM to UBP310 bound state. We observe that receptor subunits A and D swing laterally by ~19.5–21 Å at the ATD level and ~9.7–12 Å at the LBD-S1 to accommodate this asymmetric arrangement of LBD in UBP310 bound state. While smaller movements of ~3.9 to 5.8 Å, are observed in S2 lobe (Fig. 4c–e). On the other hand, subunits B/C that go from monomeric to dimeric LBD configuration swing laterally by ~20.9–22.5 Å at the ATD level. Interestingly, the separation between COMs for the B and C ATD remains unchanged between SYM and UBP310 bound forms suggesting a rigid body movement of the entire ATD layer (Fig. 4f–h; Supplementary Movies 1 and 2). The changes are more pronounced and asymmetric in the LBD layer for B/C subunits where the LBD-S1 lobe in subunit B translates horizontally by 21.6 Å while the subunit C moves only by ~9.5 Å. Further, the S2 lobes move towards the center by 23.6 Å and 2.7 Å, respectively. As a result of this, the separation between S1 lobes in B and C reduces from 51.3 Å to 35.2 Å and that for S2 lobes reduces from 37.2 Å to 25.8 Å (Fig. 4f–h). The restricted movement of LBD for distal subunit C and broader movements of proximal subunit B leads to BC dimer formation. We also compared the distal A/C and proximal B/D pairs (Supplementary Fig. 11) that show similar movements for ATD domains between 19.5 Å to 22.3 Å. The LBD domains of distal subunits A/C move by ~9.7 Å while that for proximal B/D move by 21.6 Å for B and only 12.1 Å for subunit C, again highlighting the asymmetric arrangement of LBD in UBP310 bound state (Supplementary Movie 3).

Owing to rearrangements at the LBD layer, there is also a substantial reorganization of LBD-TM linkers to go from desensitized to resting state. Consistent with a 4-fold symmetric arrangement of LBDs in the desensitized state, LBD-helix E separation in both distal A/C and proximal B/D subunit pairs is equal at ~21 Å (Supplementary Fig. 12a,b). Also, helix-E is placed ~4 Å lower in proximal B/D subunits when compared to distal A/C protomers (Supplementary Fig. 12c). In contrast, helix-E in UBP310 bound receptor has a separation of ~22 Å for A/C pair similar to that in SYM complex but ~45 Å for the B/D subunits. Further, due to the formation of BC dimer at the LBD layer, the helix-E in subunit B moves lower in vertical plane increasing the distance between helix-E from subunits B and C to ~12 Å. Interestingly, the LBD helix-E from subunits A and D align in the same plane in contrast to SYM complex where they are separated by ~4 Å (Supplementary Fig. 12). These conformational changes in helix E-M3 seem to be necessitated because of the asymmetric architecture of UBP310 bound receptor. However, the position of helix-E is based on rigid body fits of the LBD domains into the low-resolution map and hence the distances reported here are only approximate.

### N-glycans at the ATD-LBD interface

**Figure 5:**
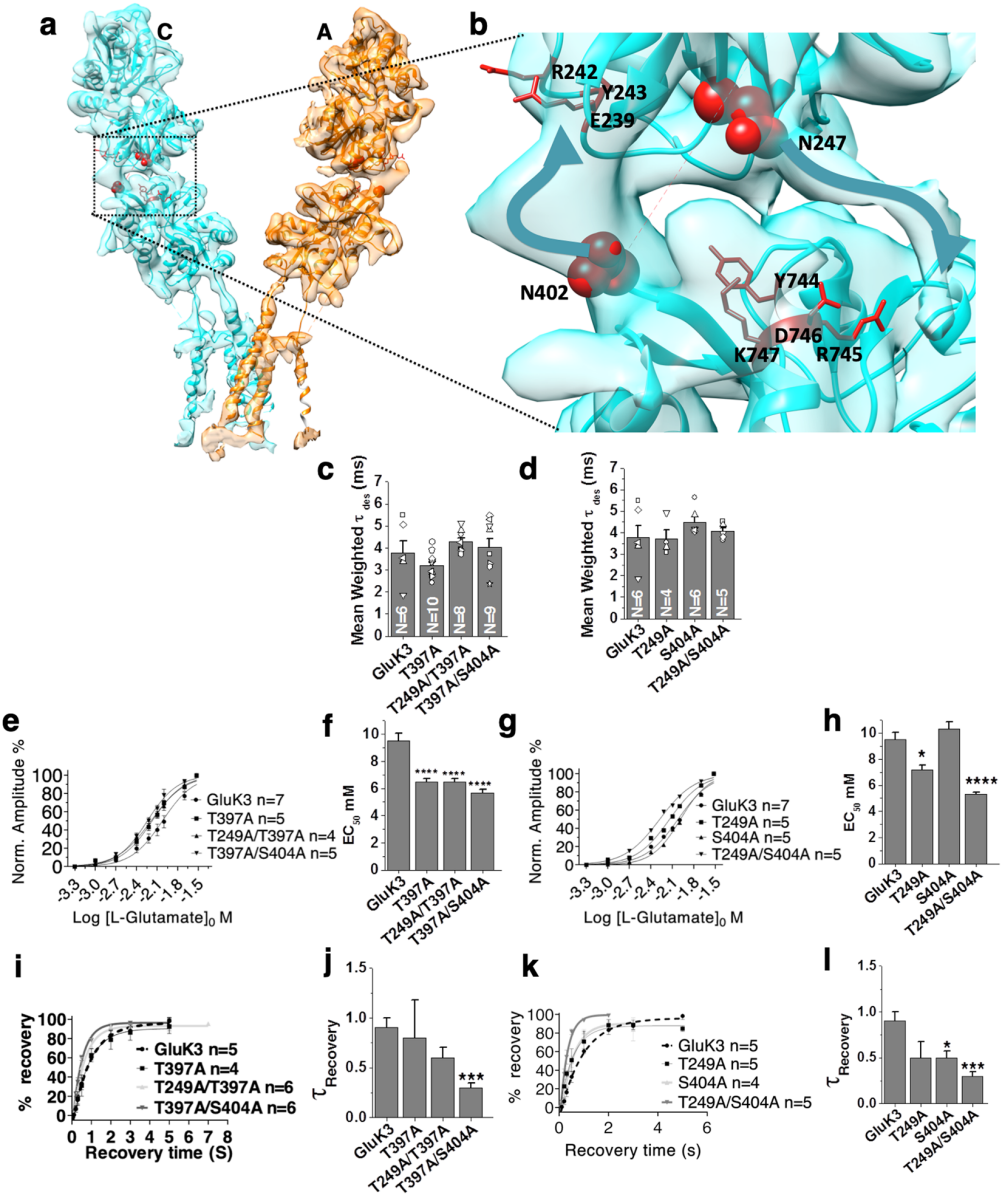
N-glycans modulate GluK3 gating properties. Panel **a** depicts the segmented density map for proximal subunits A and C fitted with the atomic model. Zoomed view of the residual density at the ATD-LBD interface for subunit A (marked by black dashed lines) is shown in (**b**) where sites for potential N-glycosylation at Asn 247 and 402 are shown in red spheres. Putative interacting residues that lie in close proximity to N-glycans are shown in red stick representation. Thick blue arrow highlights the residual density. (**c**–**l**) Show electrophysiological characterization of the various N-glycan knockouts. Panels c,d show desensitization rates (τ_des_) for the single and double glycans mutants as indicated. Mean weighted Tau (τ_des_) values obtained from a bi-exponential fit for curve obtained by 100 ms application of 30 mM glutamate are plotted for wild type GluK3 and various mutants. Panels e–h show concentration-response curves evoked by 100 ms application of glutamate at concentrations ranging from 30 µM to 30 mM normalized to the 30 mM glutamate-evoked current amplitude. Panels i–l show two‐pulse glutamate (30 mM) recovery experiments for the indicated mutants and wild type GluK3 receptors recombinantly expressed in HEK293 cells. The amplitude of the second glutamate application in a two‐pulse experiment is reported as a normalized percentage of the first glutamate application and is plotted against interpulse intervals. Recovery rates (τ_rec_) were calculated with a single exponential association fit. Error bars indicate the SEM and P values < 0.05 were considered statistically significant and are reported (*p < 0.05, **p < 0.01, ***p < 0.001, ****p < 0.0001).

iGluRs are post-translationally modified with multiple N-linked glycans that are studded on both the extracellular ATD and LBD domains^17,37^. Many of these glycans reside at the ATD-LBD interface and on the linkers connecting the two domains (Supplementary Fig. 1). They have previously been shown to modulate gating properties^37,38^ and trafficking of iGluRs^24,39^ or interactions with other synaptic proteins^40^. In GluK3 desensitized EM map, we observed residual density at the ATD-LBD dimer interface, which was not satisfied by the fitting of the ATD and LBD co-ordinates. Interestingly, the residual densities are in close proximity to potential N-linked glycosylation sites (Fig. 5a,b). In particular, the density near Asn 247 in ATD and Asn 402 in LBD appear more prominent in distal subunits as compared to proximal subunits (Fig. 5b). Similar extra density was also observed in the GluK2 (5KUF) EM map reported recently to a resolution of 3.8Å^31^. It is important to note that there is 2-fold symmetry of the ATD layer at the dimer of the dimer interface and 4-fold symmetry at the LBD layer in a desensitized receptor. Due to this symmetry mismatch, the bottom of ATD and top of the LBD domains of only the distal subunits are in close apposition and interact with each other unlike the ATD and LBD domains of the proximal subunits. Further, the N-glycans are highly flexible molecules^41^, thus they are likely to contribute poorly to EM density unless stabilized by glycan-glycan or glycan-protein interactions. Hence, the presence of this residual density at positions corresponding to N-glycan likely indicates protein-glycan interactions. It is interesting to note that N-glycans at Asn 247 and Asn 402 are suitably placed to mediate inter-domain interactions between ATD and LBD. N-glycan at Asn 247 from ATD seems to project down towards LBD and lies near it; while glycan from LBD Asn 402 is oriented towards ATD and might interact with its lower lobe (Fig. 5b).

### Disrupting the N-glycan interactions affect GluK3 gating properties

To test whether these glycan interactions could affect gating properties of GluK3 receptors, we systematically mutated N-glycan sites; first individually and then in combination. Further, we carried out electrophysiological assays measuring key gating properties like rates of desensitization, recovery from desensitized state and EC_50_ values for agonist glutamate. The N-glycan knockouts at Asn 247, Asn 395 and Asn 402 were generated in wild-type GluK3 by mutating serine or threonine residues of the consensus N-glycosylation motif to alanine. Our analysis of desensitization rates for the various mutants showed that although the mean weighted tau values slowed down when compared to wild type, they were not statistically significant (Fig. 5c,d; Table 1). However, this trend in slow down of desensitization rates is similar to that observed for GluK2 receptor triple N-glycosylation deletion at corresponding positions to GluK3 (Asn 350, Asn 395 and Asn 402) and was also shown to slow down the rate of receptor desensitization^37^.

Next, we tested whether these glycans affect the sensitivity of the receptor for the agonist by measuring dose-response curves. Indeed, our results revealed that the dose-response curves for GluK3 N-glycan mutants are left shifted with respect to wild type receptor. The macroscopic EC_50_ (glutamate) for wild-type receptors was calculated to be 9.5 mM while it was measured to be 7.2 mM (Asn 247, *P = 0.0142); 3.7 mM (Asn 395, ****P = 0.0001); 6.5 mM (Asn 247/Asn 395, **P = 0.0022); 5.3 mM (Asn 247/Asn 402, ****P = 0.0001) and 5.7 mM (Asn 395/Asn 402, ****P = 0.0001), respectively. While for single Asn 402 mutant, it was calculated to be 10.3 mM (P = 0.7602) (Fig. 5e–h). Lower EC_50_ values and a trend of slowed desensitization rates indicate that various N-glycan mutated receptors are more efficient and sensitive in responding to glutamate application.

### GluK3 N-glycan mutants recover faster from the desensitized state

We next evaluated the recovery rates from desensitization by generating recovery currents via two applications of 30 mM glutamate at different time intervals (two-pulse protocol) ranging from 50 ms to 5 s. We observe that all the GluK3 N-glycan mutants recovered from glutamate-evoked desensitized state faster than the wild type receptors with a τ_rec_ of 0.5 s (Asn 247) (unpaired t-test, P = 0.0870); 0.8 s (Asn 395, P = 0.8067); 0.5 s (Asn 402, *P = 0.0201) respectively for single knockouts and 0.6 s (Asn 247/Asn 395, P = 0.0694); 0.5 s (Asn 395/Asn 402, **P = 0.0047) and 0.3 s (Asn 247/Asn 402, ***P = 0.0007) for double glycan mutants. In contrast, the recovery rate of wild-type GluK3 had a τ_rec_ value of 0.9 s (Fig. 5i–l; Table 1). Thus, the N-glycan mutants for positions 247, 402, 395/402 and 247/402 recover ~1.8 to 3.0-fold faster than the wild type receptor, highlighting the importance of N-glycan at these positions for the receptor function (Table 1). In particular, the glycans at position Asn 247 and Asn 402 alone or in combination seem to play a key role in receptor recovery from desensitized state and glutamate sensitivity. We hypothesized that glycans at these positions might affect receptor properties by mediating glycan-glycan and/or glycan-protein interactions. Hence, we next focused our attention on the putative N-glycan interacting residues on the receptor surface.

### N-glycans likely mediate inter-domain interactions at the ATD-LBD interface

**Figure 6:**
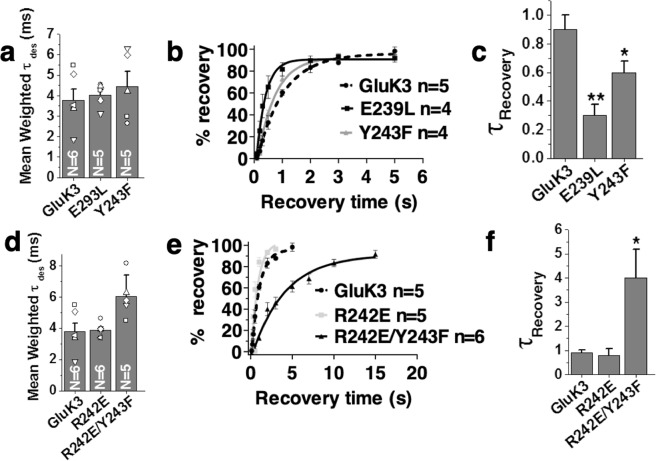
Mutations of the potential N-glycan interacting residues affect the gating properties of GluK3 receptors. Panels a–f show that mutations of residues involved in inter-domain N-glycan mediated interactions affect desensitization kinetics (**a,d**) but also the rate of recovery from the desensitized state (**b**,**c** and **e**,**f**). Disrupting these interactions slows the rate of receptor desensitization and hastens the recovery of the receptor from the desensitized state. Error bars indicate the SEM and P values < 0.05 were considered statistically significant and are reported (*p < 0.05, **p < 0.01, ***p < 0.001, ****p < 0.0001).

It has been shown that the composition, content, and length of N-glycans can be variable for iGluRs depending on the context of their expression. This in combination with the flexibility of N-glycans makes it impossible to predict the residues that might interact with them. Thus, we focused only on the residues that likely lay in close apposition to the N-acetyl glucosamine (NAG) residues of the oligo-mannose core at the ATD-LBD interface shown in Fig. 5a,b and mutated the potential interacting residues to their counterparts in GluA2 receptor. The core NAGs of Asn 402 N-glycan would likely lie in close proximity to ATD lower lobe (Fig. 5b). Interestingly, as in case of Asn 402 N-glycan knockout, single point mutants for positions Glu 239 and Tyr 243 to corresponding residues in GluA2 Leu (E239L) and Phe (Y243F) at the lower lobe of ATD slows down the receptor entry into desensitized state with a τ_des_ of 4.0 ± 0.2 ms (E239L) and τ_des_ of 4.4 ± 0.7 ms (Y243F) respectively (Fig. 6a). The mutant receptors also recover faster from desensitized state compared to wild-type with τ_rec_ of 0.3 s (**P = 0.0028) and 0.6 s (*P = 0.0465) for E239L and Y243F respectively. This is similar to the phenotype observed in case of N-glycan knockout at position Asn 402 (Fig. 6b). Interestingly, introducing a negatively charged residue by mutating arginine at position 242 to glutamate (R242E) in Y243F background (double mutant R242E/Y243F) showed a considerable decrease in the rate of desensitization (τ_des_ of 6.0 ± 1.3 ms). However, it had a slower recovery from the desensitized state (τ_rec_ of 4.0 s; *P = 0.0328) in contrast to faster recovery rates observed for knockouts and other putative interacting residue mutants (Fig. 6c,d). The double mutant R242E/Y243F recovers ~6.6 fold slower than Y243F mutant receptors and ~4.4 fold slower than wild type GluK3. However, mutant R242E alone does not alter the measured receptor properties significantly and has a τ_des_ of 3.9 ± 0.2 ms and τ_rec_ of 0.8 s similar to wild type receptor (Fig. 6c,d). We don’t fully understand the reason for this observation but it’s likely due to modulation of protein-N-glycan interactions. Owing to 2-fold symmetry at ATD dimer and dimer of the dimer interface, these mutations in a homotetrameric receptor in proximal subunits would lie close to the ATD dimer of the dimer interface. However, they are away from the central axis of the receptor’s tetrameric interface and are not likely to perturb tetrameric assembly. Thus, the effects seen are likely due to modulation of N-glycan-protein interactions on the distal subunits.

To determine if the non-functional mutants were expression or trafficking deficient, we analyzed the surface expression of all the mutants by biotinylation assay. It was found that all the mutants were able to reach the cell surface, albeit in variable amounts. Surprisingly, N721A glycan knockout failed to reach the cell surface (Supplementary Fig. 13). This N-glycan site is present at the LBD-TMD interface is reported to be glycosylated in LBD expressed in insect cells^42^. Interestingly, it is conserved in all the kainate receptor subunits (GluK1-GluK5) but absent in NMDA and AMPA receptors. This suggests that glycosylation at this site might be essential for the assembly and trafficking of kainate receptors. However, it needs to be explored further to elucidate its role fully.

Next, we investigated the potential interacting residues for Asn 247 N-glycan. The core NAGs of Asn 247 N-glycan likely lie in close proximity to S1 lobe of LBD. We checked double mutants Y744L/R745G and quadruple mutants Y744L/R745G/D746T/K747P for activity. However, in spite of reaching the cell surface (Supplementary Fig. 13), we could not record measurable currents from these receptors. Despite this, our glycan knockout assays highlight the importance of Asn 247 glycan in the modulation of receptor functions.

To summarize, our structural and functional data suggest potential inter-domain interactions mediated by N-glycans, the perturbations of which alter receptor functions. It is noteworthy that the disruption or changes in interactions of N-glycans at position Asn 247 and Asn 402 that potentially mediate the ATD-LBD inter-domain interactions have maximum effect on the receptor properties. Our study highlights the contribution of N-glycans in enhancing the stability of the desensitized state and thus slowing down the recovery from the desensitized state.

## Discussion

**Figure 7:**
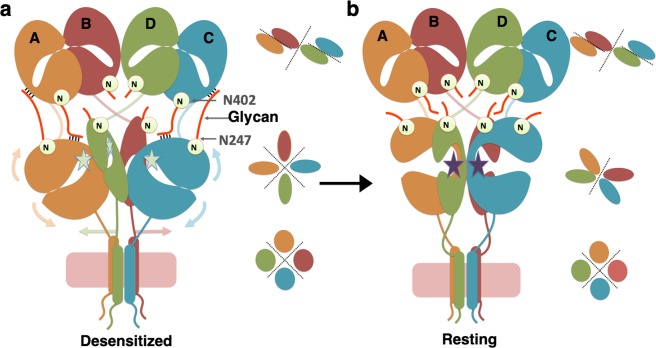
Model depicting the effect of N-glycans on the receptor recovery from the desensitized state *via* interdomain interactions at the ATD-LBD interface. Inter-domain interactions at the ATD-LBD interface mediated by N-glycans (thick red lines) at Asn 247 and Asn 402 positions are shown in a desensitized GluK3 receptor in panel a. Panel b shows a schematic for “fully-recovered” receptor where the N-glycan mediated interactions (depicted in black lines) would likely be disrupted due to rearrangements at the LBD layer. These interactions likely contribute to impeded receptor recovery from the desensitized state since the desensitized to resting state transition requires that LBD domains return to dimeric configuration. These transitions would restore 2-2-4 symmetry for ATD, LBD and TM domains respectively in fully-recovered state (**b**) from 2-4-4 symmetry observed in the desensitized state (**a**) shown in the cartoon for various domains.

Current advances in the field of ionotropic glutamate receptor biology indicates that KA receptors are involved in multifunctional neuronal activity and have a profound role in health and diseases^2,43–45^. Using a multi-pronged approach, combining cryo-EM, X-Ray crystallography, electrophysiology, we studied GluK3-kainate receptor structures trapped in the desensitized and resting/closed states. Primarily, three major states exist in the gating cycle of glutamate receptor ion channels namely; resting, activated and desensitized state. Binding of agonist to the resting state receptor leads to a short-lived active state, which immediately relaxes to a desensitized state to relieve the strain caused by the activated state onto linkers between LBD and TM^29,34^. It quickly rearranges to a more energetically favourable desensitized conformation, where the LBD acquires the quasi 4-fold symmetry. Removal of agonists or binding of antagonists in *in-vitro* conditions allows the receptor transition to resting/closed state by rearranging LBD to a dimeric state, which leads to re-positioning of the linkers between LBD and TM^31^. It is well established that kainate receptors recover slower than AMPA receptors^46^. Also, the desensitized state in kainate receptors is ~100 fold more stable than their AMPA counterparts^46^. N-glycans by virtue of their large size, position, and chemical composition could mediate both intra- and interdomain interactions within the same subunit or other subunits in a tetramer. N-glycosylation corresponding to GluK3-Asn 402 in GluN1 (Asn 440) contributed to the stability of closed clamshell LBD structure^47^. In this simulation study on isolated LBD, it was suggested that hydroxyl groups of N-glycan mannose are likely to interact with hydrophilic residues present on LBD only in the closed conformation. While intra-domain LBD interactions were shown, no predictions for inter-domain interactions could be made as the investigation was limited to isolated LBD domains and hence they cannot be precluded. These inter and intra domain interactions would potentially modulate receptor functions. Our GluK3-SYM and GluK3-UBP310 complex structures combined with electrophysiology-based functional assays; identify structural elements on receptor surface, which potentially interact with N-glycans at Asn 247 and Asn 402 near the ATD-LBD interface (Fig. 7). These interactions likely impede receptor recovery from the desensitized state since the desensitized to resting state transition requires large-scale movements of the distal and proximal LBD domains to regain the dimeric configuration. The distal domains are stabilized in the desensitized state likely by inter-domain interactions mediated by N-glycans at Asn 247 and Asn 402 apart from other protein-protein interactions. Our results provide a plausible explanation for the low potency of glutamate, which might be due to a partially recovered resting state in GluK3, reducing the efficacy of bound ligand in channel opening. This could also explain why the GluK3 receptors might desensitize faster even at sub-saturating glutamate concentrations consistent with the function of GluK3 receptors at the mossy-fiber CA3 hippocampal cell synapse^11^. Disruption of the N-glycan mediated interactions likely allows full-recovery leading to slower desensitization, higher glutamate potency and faster recovery from the desensitized state. However, this needs to be established further by more focused electrophysiology experiments. Further, in the context of heteromeric receptors formed between GluK1-GluK3 receptors, our findings remain valid as both N-glycosylation sites and potential interacting residues are conserved (Supplementary Fig. 1). Similarly, for the receptors containing the “high-affinity” GluK4 and GluK5 subunits, the residues corresponding to E239 and Y243 are aspartate and glutamate/aspartate respectively for the two sites in GluK4 and GluK5 subunits and may still interact with the N-glycan corresponding to Asn 402 on heteromeric assembly with GluK1-GluK3 subunits. Further, the interactions mediated by N-glycans would also affect the functional modulation by auxiliary proteins like Neto1 and Neto2 and has been shown earlier for GluK2^20^ but needs more exploration in case of GluK3 receptors.

## Methods

### Construct design

We initially tried expression of the full-length rat GluK3 subunit containing point mutants R591Q (Q/R site) but this construct had very low expression and poor profile on size exclusion chromatography (SEC) and hence was not suitable for structural analysis. To achieve better surface expression, the native signal peptide of GluK3 was replaced with that of GluK2 and was sub-cloned into the pEGBacMam vector^48^ for baculovirus-based expression in mammalian cells using standard molecular biology techniques. To screen constructs via fluorescence detection^25^ and for affinity purification, a thrombin recognition site along with linker sequence (GLVPRGSAAAA) was inserted between GluK3 and the coding sequence for the A207K non-dimerizing EGFP mutant, with a C-terminal octa-histidine (His8) affinity tag. To improve solubility and stability, the GluK3 construct was truncated at C-ter ∆826 and cysteine residues at positions 86, 305 were mutated to threonine while cysteine 547 was replaced with valine. This construct referred as GluK3_EM_ had good expression and tetrameric receptor profile as screened via FSEC and hence was chosen for large-scale expression and purification.

### Whole-cell voltage-clamp recordings

To test the functionality of GluK3 wild type, GluK3_EM_ construct, and various mutants, we performed whole-cell patch clamp recordings. Passage 20–35 human embryonic kidney 293 (HEK 293) cells were cultured on coverslips placed in a 35-mm polystyrene dish. Cells were transiently transfected either with GluK3_EM_ or co-transfected with Wild type or mutant receptors along with GFP expressing plasmid (2 μg/dish) using Xfect reagent (Clontech). Currents were recorded from medium-sized cells expressing a moderate level of fluorescence from either the fused EGFP in case of GluK3_EM_ or co-expressed EGFP and having a capacitance of ~5–6 pF at 48–60 hours post-transfection. Whole-cells were held under voltage clamp at −80 mV using a HEKA USB 10 amplifier. Ultrafast ligand application was achieved by using a two-barrel theta pipette ultra-fast perfusion system mounted on a piezoelectric device (Multichannel systems) controlled by Patchmaster software (HEKA). Responses were filtered at 3 kHz and digitized at 20 kHz. Recording pipettes were pulled (Sutter, P-1000) from borosilicate glass capillaries (1.5 OD × 1.17 × 100 L mm, Harvard Apparatus) and fire polished with an in-house developed microforge to reduce the tip diameter and get pipettes with resistance between 2–3 Mega ohms. Pipettes were filled with intracellular solution (ICS) containing 30 mm CsCl, 100 mm CsF, 4 mM NaCl, 10 mm HEPES, 5 mm EGTA, 2 mM Na_2_ATP and 0.5 mm CaCl_2_, pH 7.2 and osmolarity ranging between 290–300 mosmol/L. Cells were continually perfused with extracellular solutions (ECS) containing 150 mm NaCl, 2.8 mm KCl, 10 mm HEPES, and 0.5 mm CaCl_2_, pH 7.3 and osmolarity ranging between 295–305 mosmol/L at a flow rate of ~0.2 ml/min. After attaining whole-cell voltage clamp, cells were raised off the dish and positioned near the interface of the theta pipette solution streams. One stream contained control solution (ECS) while the other stream-contained ligands (l-glutamate, SYM or UBP310 at indicated concentrations) dissolved in the control solution. Longer applications of ligand i.e. 100 ms were perfused to measure the whole-cell desensitization kinetics. The whole cell recordings were acquired using Patch master V2X90.2 (Heka Elektronick) 3 min after establishment of the whole-cell configuration. Raw data files were exported into Igor pro (ITX) and converted into abf files, compatible for pClamp by using ABF Utility. The macroscopic rate of desensitization (*τ*_des_) was measured by the exponential fit to the decay of current from ~90% of its peak amplitude (*I*peak) to baseline. The desensitization kinetics were fitted by using the single exponential, 2-term fitting (Levenberg-Marquardt). Mean weighted *τ*_des_ values were calculated and are reported. However, it should be noted that slower solution exchange time with the use of whole cells may limit resolution of the very fast desensitization events of GluK3 receptors. For recovery from desensitization, a paired-pulse protocol was used and the amplitudes of the test pulse responses were normalized to the amplitude of the desensitizing pulse response (calculated as percentage recovery); and plotted against the interval between desensitizing and test pulses. The time courses of recovery from desensitization were fit with a one‐phase association exponential function. Dose-response experiments were performed with different concentrations of glutamate in the range of 30 µM to 30 mM. Dose-response curves were plotted as the percentage of maximal response against Log [L-Glutamate] concentrations and fitted with Hill equation in Prism, version 8.0.1 (GraphPad Software).

### Statistical analysis

Comparisons between conditions were made by unpaired *t* test (two-tailed) or by one-way ANOVA followed by Dunnett’s multiple comparison. Statistical analysis was carried out in Prism, version 8.01 (GraphPad software). P values < 0.05 were considered statistically significant and are reported (*p < 0.05, **p < 0.01, ***p < 0.001, ****p < 0.0001).

### Expression and purification of GluK3 receptor

GluK3_EM_ was transformed into DH10Bac cells to prepare bacmid for baculovirus generation following standard protocol^48^. HEK293 GnTI^−^ cells at a density of 2.0–3.5 × 10^6^ cells/ml were infected with P2 virus at a multiplicity of infection (MOI) value of ~1. After 20 hours of incubation, 10 mM sodium butyrate was added to the culture flasks and transferred to 30 °C for protein expression. Cells were harvested 70 hours post infection and stored at −80 °C until further processing. Cell pellets were resuspended in ice-cold buffer (25–30 mL/L) containing 150 mM NaCl, 20 mM Tris (pH 8.0) and 1X Protease Inhibitors Cocktail (Roche). The resuspended cells were disrupted by ultrasonication (QSonica sonicator, 4 cycles of 90 sec (15 sec on/ 15 sec off) with power level 7 using medium size probe) with constant stirring. Care was taken to maintain the temperature of cell suspension below 12 °C. The lysate was clarified by low-speed centrifugation and membranes were collected by ultracentrifugation (37,000 rpm, 60 min). Membrane pellets were homogenized and solubilized for 45 min in buffer containing 150 mM NaCl, 20 mM Tris (pH8.0), 29.5 mM n-dodecyl-β-D-maltopyranoside, and 6 mM cholesterol hemisuccinate at 4 °C. Insoluble material was removed by centrifugation (40,000 rpm, 60 mins) and cobalt-charged TALON metal affinity resin (~7 mL) was added to the supernatant together with 10 mM imidazole to allow batch binding for 3 hours at 4 °C. The beads were packed in the column, washed with 10 mM and 40 mM imidazole containing buffer (20 mM Tris, 150 mM NaCl, 0.75 mM n-dodecyl-β-D-maltopyranoside, 0.03 mM cholesterol hemisuccinate) until the OD at 280 nm reached close to zero and then bound receptors were eluted with buffer containing 250 mM imidazole. Peak fractions were pooled and concentrated to ~1.9 mg/ml and digested overnight at 4 °C with thrombin at a 1:100 wt/wt ratio. GluK3 receptor tetramers were isolated by gel filtration chromatography (Superose 6 10/300) in a buffer containing 150 mM NaCl, 20 mM Tris (pH 8.0), 0.75 mM n-dodecyl-β-D-maltopyranoside, and 0.03 mM cholesterol hemisuccinate were concentrated to ~1.7 mg/mL (MWCO 100 kDa). The final n-dodecyl-β-D-maltopyranoside and cholesterol hemisuccinate concentration calculated based on fold reduction of protein volume during concentration was ~3.5 mM and 0.15 mM respectively.

In order to trap the purified receptors in different states, they were incubated with ligands directly before imaging. 2S, 4R-4-methyl glutamate (SYM) at a final concentration of 2 mM was added to the protein to trap desensitized state whereas, 100 µM UBP310 was used to stabilize the receptor in resting state. Screening of the concentration and effect of these agonists and antagonist onto the stability of the receptor was checked by FSEC analysis (data not shown).

### Specimen vitrification and Cryo-electron microscopy

For both GluK3-SYM and UBP310 complex, Quantifoil R1.2/1.3 Au 300 mesh grids were glow discharged for 90 secs at 15 mA. 2.5 µl of protein at 1.7 mg/ml was applied to the grid thrice followed by blotting for 5 s at 100% humidity, 4°C and vitrified by plunging into ethane cooled by liquid nitrogen using vitrobot. The grids were clipped and loaded into a 300 kV Titan Krios microscope equipped with Falcon 3 direct-detector camera. Images were recorded in super-resolution counting mode with a nominal magnification of ~59,000X and defocus range of −2.3 to −3.3 µm in steps of 0.3. Total 25 frames were collected in a movie with 60 s exposure, and a dose rate of 1.07 e/Å/s with a pixel size of 1.37 Å for SYM and dose rate of 1.17 e/Å/s with a pixel size of 1.41 Å for UBP310 complex respectively.

### Electron microscopy image processing and model building

All the images (719 images for GluK3-SYM and 1693 for GluK3-UBP310) were subjected to beam-induced drift correction using UCSF MotionCor2^49^ followed by CTF estimation via Gctf ^50^. Manual curation was carried out to remove micrographs with considerable ice contamination or poor CTF fits. RELION 2.1^51^ was used to pick ~1000 particles manually and subjected to reference-free 2D classification. Selected 2D classes from this step were used as a reference for automated particle picking from entire datasets. Autopicked particle stacks were exported to cryoSPARC^52^ for GluK3-SYM and cryoSPARC v2 for GluK3-UBP310 to carry out 2D classification. Data were cleaned up *via* iterative rounds of 2D classification and subsequent removal of classes with unclear features, ice contamination or carbon. Initially, 77276 particles selected for GluK3-SYM and 138369 particles for GluK3-UBP310. After successive rounds, 14648 and 54001 particles were chosen for the GluK3-SYM and GluK3-UBP310 respectively. These were used to generate reference free *abinitio* 3D reconstruction. An additional cycle of 2D classification and removal of poorly defined particles selected 9730 particles that were utilized for 3D classification and refinement for SYM bound receptor in C1 symmetry. Homogenous refinement as implemented in cryoSPARC resulted in ~7.4 Å resolution map according to gold standard Fourier Shell Correlation (FSC). For GluK3-UBP310, 3D reconstruction and homogeneous refinement by imposing C1 symmetry lead to ~8 Å resolution map. Sorted stack of 38853 particles was subjected to per particle local motion correction followed with 2D classification and *abinitio* reconstruction into two 3D classes in cryoSPARC V2. The best 3D class (27194 particles) was further subjected to homogenous refinement resulting in a map with a resolution of ~8.3 Å. This was followed with the non-uniform and local refinement that improved the map resolution to ~ 8.1 Å and ~7.7 Å (0.143 FSC) respectively. Final maps for both the complexes were sharpened and ResMap^53^ and Local Resolution module as implemented in cryoSPARC workflow was used for estimation of local resolution.

### Crystallization and structure determination of GluK3 LBD with SYM

GluK3 LBD S1S2 domain from residues N402-K515 of S1 domain linked by GT linker to P638-P775 of S2 domain (numbering according to mature polypeptide) was cloned into a pET22b vector with an N-terminal hexahistidine tag (MHHHHHH) followed by LVPRGS thrombin cleavage site. Protein was expressed in *E. coli* Rosetta Gami 2 (DE3) strain and purified by following the previously published protocol^12^. Purified and concentrated protein was dialyzed against agonist SYM (0.5 mM) containing buffer. The final protein at a concentration of ~19 mg/ml in buffer containing 20 mM HEPES pH 7.0, 200 mM NaCl, 5 mM Zinc acetate, 1 mM EDTA and 500 µM SYM was used for crystallization trials at 22 °C. Best crystals grew in 50 mM HEPES, pH 7.0, 4% PEG 8000, 100 mM NaCl and were cryoprotected by transferring to a solution containing 25% glycerol as cryoprotectant along with all the components of protein and crystallization mother liquor. X-ray diffraction data were collected at the European Synchrotron Radiation Facility (ESRF), France. The data were indexed, scaled and merged using iMosflm^54^ and SCALA^55^. Finally, the structure was solved by molecular replacement using crystal structure of LBD-Kainate complex (PDB: 3U92) as a template with PHASER^56^ and refined using Phenix^57^ to a final resolution of 1.83 Å (Supplementary Table 1). Structural analysis and figure preparation was carried out using UCSF Chimera^32^ and PyMOL^58^. Bound SYM was well resolved in our crystal structure along with interacting residues (Supplementary Figs 14 and 15). Structure of GluK3-SYM LBD is very similar to GluK2-SYM structure reported earlier with r.m.s.d of 0.78 for superimposition of Cα atoms (Supplementary Fig. 16).

### Model building of the full-length receptor

Model for the GluK3-SYM tetramer was built by rigid body fitting of individual domains into EM map in UCSF Chimera. Four copies each of GluK3 amino-terminal domain ATD (PDB code, 3OLZ) and ligand binding domain (GluK3-LBD-SYM complex crystal structure determined in this study) was used. Further, for GluK3 TM, the sequence was threaded onto the transmembrane domain of GluK2 (PDB code: 5KUF) and was used for fitting into EM density. For GluK3-UBP310, PDB: 3OLZ used for ATD whereas LBD and TM were threaded onto LBD of 5CMK (LY466195 bound monomer) and TM of 5KUF respectively. Coot^59^ was used to jiggle fit coordinates into EM density followed by multiple rounds of real space refinement (rigid body) of the complete model using PHENIX^33^. All the four subunits fit well into the EM map of both GluK3-SYM and GluK3-UBP310 shown in Supplementary Figs 7 and 8 with a definite density of ATD, LBD, and TM (pre-M1, M3 and M4) segments.

### Site-directed mutagenesis

All the mutations were made in wild type GluK3 receptors following the standard protocol for site-directed mutagenesis and confirmed by sequencing of the entire GluK3 coding region. To disrupt N-linked glycosylation, S/T was substituted to alanine in N-linked consensus glycosylation sequences (NXS/T). Thus, T249, T397, S404, and T723 were mutated to alanine to disrupt N-linked glycosylation at N247, N395, N402, and N721 respectively. To check the putative N-glycan interacting residues for N247 glycans, residues Y744, R745, D746, K748 were mutated to the corresponding residues in GluA2. Y744L/R745G (double mutant) and Y744L/R745G/D746T/K748P (quadruple mutant) were also generated. Similarly, mutants E239L, R242E, and Y243F were made to test potential N402 glycan interacting residues.

### Surface biotinylation assay

To evaluate the surface expression of mutant receptors we carried out surface biotinylation assay. HEK293 cells were seeded in 6-well plates and transfected with various mutants along with wild type GluK3. After 40 hours of transfection, cells were washed with 1 ml ice-cold PBS/CaCl_2_/MgCl_2_ buffer twice. Following which, they were treated with 0.3 ml of 0.5 mg/ml Sulfo-NHS-SS-biotin solution for 30 min at 4 °C. Post incubation, biotin solution was removed and the reaction was stopped by addition of biotin-quenching solution (50 mM glycine in PBS/CaCl_2_/MgCl_2_ buffer). This was followed by three rounds of washing with ice-cold PBS buffer. Cells were lysed in 100 μl of solubilization buffer (150 mM NaCl, 20 mM Tris, pH 8.0, 1 mM PMSF), centrifuged to remove debris. The supernatant was mixed with 50 μl of 50% slurry of neutravidin agarose beads and rotated for 5 hours at 4 °C to allow binding of biotinylated proteins. Following this, the beads were washed extensively for removal of unbound proteins. Post final wash, the supernatant was removed and 25 μl of 4x SDS loading buffer was added to the beads and heated for 5 mins at 95 °C. Eluted proteins were resolved via SDS-PAGE, electroblotted onto PVDF membrane and probed with Anti-GluR6/7 monoclonal Antibody (Sigma) to identify surface expressed wild type and mutant GluK3 receptors. (Supplementary Fig. 16).

### Statistics

No statistical methods were used to predetermine the sample size. The experiments were not randomized, and the investigators were not blinded to allocation during experiments and outcome assessment.

## Supplementary information

SUPPLEMENTARY INFO

Supplementary movie 1

Supplementary movie 2

Supplementary movie 3

## Data Availability

The cryo-EM density reconstructions and final models were deposited with the Electron Microscopy Data Base (accession code EMD-9821 and EMD-9822) and with the Protein Data Bank (accession code 6JFY, 6JFZ). PDB code for coordinates and structure factors for GluK3 ligand binding domain complex with SYM is 6JMV. All other relevant data supporting the key findings of this study are available within the article and its Supplementary Information Files or from the corresponding author upon request.
